# Characterising subjective cognitive decline: Insights from an at‐risk research clinic population

**DOI:** 10.1002/alz70860_106136

**Published:** 2025-12-23

**Authors:** Muthoni Gichu, Alison Canty, Cassandra Thomson

**Affiliations:** ^1^ University of Tasmania, Hobart, Australia, TAS, Australia; ^2^ University of Tasmania, Hobart, TAS, Australia

## Abstract

**Background:**

Subjective cognitive decline (SCD) is a potential early marker of dementia, yet its characterisation remains understudied. This study explores the demographic, clinical characteristics, medical history, lifestyle factors and psychological profiles of individuals with SCD, focusing on the 14 risk factors by the Lancet commission on dementia prevention, intervention and care.

**Method:**

Using a cross‐sectional data from a research cognitive clinic, 97 individuals with a diagnosis of SCD, assessing risk factors including hypertension, diabetes, smoking, obesity, traumatic brain injury, excessive alcohol consumption and sleep quality.

**Result:**

Demographically, the cohort was predominantly female (67%) with an average age at diagnosis of 66.09 years (SD=8.3, range 44‐85) and 84% having completed secondary education. Clinically, 42% reported memory complaints, while 39%, 35% and 34% described attention, social or language difficulties respectively. Medical history revealed various prevalence of hypertension (36%), high total cholesterol (25%), high Low‐Density Lipoprotein (LDL) level (20%), Traumatic Brain Injury (26.8%), obesity (35%) diabetes (5%), polypharmacy (31%), 54% and 35% reported visual and hearing impairment respectively, and 79% reported poor sleep quality. Lifestyle factors showed 25% were current smokers, 18% had a history of excess alcohol consumption. Psychological factors were widely variable with 50% screening positive for depression, 8% for anxiety and 4% for high stress levels.

**Conclusion:**

Modifiable factors particularly poor sleep quality, depression, obesity and hypertension are prevalent in this SCD cohort. These findings highlight the need for targeted interventions addressing the risk factors to mitigate dementia risk in this at‐risk populations.

